# Managing geese with recreational hunters?

**DOI:** 10.1007/s13280-018-1070-7

**Published:** 2018-07-03

**Authors:** James Henty Williams, Thorsten J. S. Balsby, Helle Ørsted Nielsen, Tommy Asferg, Jesper Madsen

**Affiliations:** 1 Department of Bioscience, Aarhus University, Kalø, Grenavej 14, 8410 Rønde, Denmark; 2 Aarhus University, Bartholins Alle 7, 8000 Aarhus C, Denmark

**Keywords:** Adaptive management, Behaviours, Geese, Harvest management, Hunters, Motivations

## Abstract

As many goose populations across the northern Hemisphere continue to rise, the role of hunters to manage these populations is increasingly being considered. We studied recreational goose hunters in Denmark to assess their behavioural and motivational characteristics, willingness to alter their hunting effort, as well as their ability to act as stewards of a rapidly increasing goose population. We identified several behavioural characteristics that typify effective goose hunting practices. We suggest a degree of specialization is necessary to increase goose harvests, as well as mitigating animal welfare issues (e.g. wounding). However, the majority of Danish goose hunters can be considered to be casual participants in this form of hunting. This poses a challenge for wildlife managers wishing to engage recreational hunters to manage highly dynamic wildlife populations, such as geese. If recreational hunters are to be used as a management tool, wildlife managers and hunting organizations will need to consider how best to facilitate skill development, hunting practices and socially legitimate hunting ethics to foster the stewardship role of hunting. We conclude that it is incumbent on wildlife managers to recognize and deal with both internal factors (e.g. skill development) and external influences (e.g. animal welfare concerns). In doing so, potential tensions in the multi-functionality of hunting can be alleviated, maintain hunting as a legitimate and accepted recreational past-time and management tool.

**Electronic supplementary material:**

The online version of this article (10.1007/s13280-018-1070-7) contains supplementary material, which is available to authorized users.

## Introduction

Several goose populations in Europe have grown dramatically in size since the 1960s. These populations are increasingly the cause of conflicts with agricultural interests as well as posing threats to air safety, human and animal health and potentially detrimental effects on vulnerable ecosystems (e.g. arctic tundra) (Fox and Madsen 2017). Progressively the management of problematic goose species is being considered at a population level, employing adaptive harvest management (AHM) and requiring national and international coordination (Stroud et al. 2017). In Europe, the use of hunters and lethal control to manage problem situations involving geese has been limited and predominately confined to local schemes that are highly regulated (e.g. requiring dispensations) (Bradbeer et al. 2017; McKenzie and Shaw 2017). However, in 2012 the Svalbard population of the pink-footed goose (*Anser brachyrhynchus*) was chosen as the first European test case for the development of an International Species Management Plan (ISMP) (Madsen and Williams 2012). This management plan included setting a population target as part of a series of objectives and management actions, instigated to reduce agricultural conflicts and avoid tundra degradation (Madsen et al. 2017). To maintain a stable population size, an AHM strategy was developed using recreational hunting. The initial aim of the AHM strategy was to reduce the population to an agreed level of 60 000 birds, requiring its reduction from a peak of 80 000 (the estimated population size in 2013).

The use of hunting to manage a population shifted the emphasis of hunting as a recreational pastime to that of a management tool as part of a structured adaptive management framework. Hunting has multiple roles in contemporary society that span socio-cultural (e.g. recreation, identity, food and trophies), ecological (e.g. wildlife management) and economic functions (e.g. crop protection) (Fischer et al. 2013b). Tensions can arise between these functions of hunting, particularly if functions (e.g. economic) intended by wildlife managers are at odds with those accepted by society and its various constituent communities (e.g. recreational hunters). The acceptance of hunting as an activity as well as its use as a management tool is dependent on perceptions of why and how it is practised. Negative attitudes towards hunting can emerge if it is perceived as excessive or where the motives for hunting are perceived as simply for the sake of killing or excitement, rather than satisfying existential needs (e.g. providing food) or providing a valued service (e.g. ecological benefits) (Fischer et al. 2013a; Gamborg and Jensen 2017). Furthermore, hunters themselves have their own codes of conduct, taboos and motives that guide acceptable hunting practices (von Essen 2018). The motives of recreational hunters have been well studied for a variety of hunting types, revealing multiple motives (e.g. those seeking a nature experience, gaining food, for excitement, skill development and social bonding) (Decker and Connelly 1989; Schroeder et al. 2006; Radder and Bech-Larsen 2008). It is also evident, particularly in Scandinavian countries, that although hunters may hunt for self-fulfilment there are other rationale claimed for their pastime. Many hunters consider hunting as having a beneficial role in the sustainable management and care of wildlife and regard themselves as responsible stewards (Kaltenborn et al. 2013; Gamborg et al. 2017).

In our study, it was recognized that desired outcomes of the AHM strategy were very much dependent on the willing participation of recreational hunters. The plan was dependant on the interest and acceptance by hunters to, initially, shoot more geese (Madsen et al. 2017). However, detailed knowledge was lacking about basic hunting behaviours and motivations of recreational goose hunters in Denmark, who shot the majority (ca 80%) of pink-footed geese (Madsen et al. 2017). Denmark and Norway are the only two countries along the flyway where hunting them is permitted. We focused our attention on this community of hunters to gain a better understanding of the characteristics of different segments, as well as the potential of these segments to play an active and willing part in the management of a goose population. Initial analysis indicated that there were marked differences in annual bag sizes and we opted to create exploratory hunter groups based on total goose bag sizes for the 2013 hunting season. Our objectives for this study were to (a) assess the hunting activity of these groups, as well as what influence/impact they correspondingly had on the Danish goose harvest; (b) determine what behavioural characteristics differentiated these groups and what enabled some hunters to achieve higher goose bags; (c) identify if there were any motivational characteristics that were indicative of these groups and (d) determine the attitudes of these groups towards managing goose populations and their willingness to modify their goose hunting activity.

## Materials and methods

### Hunting bag records

We had access to the Danish wildlife bag records (Vildtudbyttestatistik) which are collected by the Danish Environment Agency and a national database processed and maintained by DCE—Danish Centre for Environment and Energy at Aarhus University. All licensed Danish hunters are legally required to report what they have shot annually via an online reporting system. We focused our analysis on data collected for three hunting seasons from 2013 to 2015. Reporting of geese for these hunting seasons was, for the first time, at species level and provided a comprehensive dataset of species-specific hunting bag records for individual Danish hunters. Each hunter record had a unique I.D. code for analysis but personal data were anonymous. We used these hunting bag records to assess the overall goose bag and hunting activity of Danish goose hunters for the 2013–2015 hunting seasons. Binomial generalized linear models (GLM) were used to test if bag size could predict the likelihood of continuing to hunt geese or change bag size groups.

### Survey

#### Sample design

We used stratified sampling to create three sample groups based on overall individual goose bags for the 2013 hunting season: low (1–2 geese); medium (3–10 geese) and high (11+ geese). Submitted goose bag records were available for 9660 hunters and were divided into the three desired sample groups. Of these, 666 hunters for each sample group were randomly selected and invited to participate in the survey. This sampling procedure was undertaken to ensure sufficient responses were gained for each sample group, in particular those shooting 11+ geese. In total, invitation letters were sent to 1998 goose hunters, throughout Denmark. They were requested to participate and complete a questionnaire using the online survey tool, SurveyXact (https://www.surveyxact.dk). An online survey was used, as in 2012 online reporting of Danish hunting bag records had been introduced. This development led to very high (98%) reporting rates, as renewals of annual hunting licences were dependent on hunters reporting their hunting bags (Asferg 2016). The survey was conducted in September 2014. Consent to participate was voluntary and was obtained by email. Participants were advised of the nature of the study and given written details of questions to be asked prior to interviews. All participants gave written informed consent to take part in the study. Anonymity and confidentiality of the interviews was guaranteed. A total of 962 hunters completed the questionnaire, with an overall response rate of 48% and a high number of respondents for each of the sample groups (Table 1).

**Table 1:** Sample structure for Danish goose hunter survey, composed of hunters who submitted hunting bag reports and shot geese in 2013. Three sample groups based on individual overall goose bags

Survey sample groups	Population	% population	Invited hunters	Respondents	Response rate (%)
Low bag group 1–2	5020	52	666	282	42
Medium bag group 3–10	3490	36	666	345	52
High bag group 11+	1150	12	666	335	50
Total	9660		1998	962	48

#### Questionnaire

The questionnaire was based on a previous survey conducted amongst Danish hunters undertaken in 2000 (Hansen 2000). This was revised and adapted after a series of qualitative interviews carried out with 9 goose hunters conducted at their homes. The questionnaire was then tested and completed online by 6 of these goose hunters. Before and after piloting, advice from hunting experts was also sought. For this study, only a selected number of questions were regarded as pertinent in order to differentiate hunters and determine characteristics that distinguished the three sample groups. The selected questions covered two broad traits of hunters that could be used to model and predict goose bag sizes: (1) goose hunting behavioural characteristics and (2) reasons for going goose hunting (motivational characteristics). All categorical questions selected had sufficient responses gained at each predictor level for analysis (> 50). In addition, three questions were used to assess respondent attitudes to managing goose populations and their willingness to alter their hunting activity (see Table 3).

### Predictors of annual goose bags

The survey questions provided the source data for our predictor variables. They were selected in order to define plausible alternative hypotheses (Burnham et al. 2011) reflecting distinct behavioural and motivational characteristics that might influence goose hunting opportunities, their success and ultimately determine annual goose bag sizes. Our selection of questions and subsequent model formulations were based on information gained from a number of published studies about recreational hunter behaviours and preferences, as well as our own understanding of goose hunting in Denmark (Kaltenborn et al. 2012; Primdahl et al. 2012; Wam et al. 2013; Lundhede et al. 2015; Jensen et al. 2016). Seven survey questions were used as potential behavioural predictor variables (see Table 2). Five questions assessed respondent motivations and attitudes towards goose hunting. These were considered as potential variables indicating respondent motivations for achieving higher or lower goose bags (see Table 3). These variables were used to formulate two sets of potential models to predict annual goose bags, which we treated as a continuous response variable: (1) behavioural models and (2) combined behavioural and motivational models.

**Table 2:** Survey questions considered as potential behavioural predictor variables

Survey question	Response type and modelled levels^a^	Predictor variable	Comments and hypothesized impact
Approximately how many days were you out hunting and you shot geese during 2013–2014 hunting season? How often were you out goose hunting without shooting geese in 2013–2014?	Open numeric Continuous	Days	Two questions combined to give total number of goose hunting days. Indicative of investment (time and effort) and the more often out hunting increases likelihood of success and larger annual goose bags
How many goose hunting areas do you have access to, including your most often used area?	Categorical 1 2 3 4+	Areas	Access to multiple hunting areas increases hunting opportunities (right place and right time) and hence higher annual goose bags
How often do you or your hunting partners check if geese are in your hunting area?	Categorical No/not often Weekly Daily	Check	Investment of time as more often hunting areas checked increases success of each hunting day (greater likelihood of geese in hunting area) and larger annual goose bags
What was the distance from your home to your most often used goose hunting area in 2013–2014?	Categorical 0–10 km 11–40 km 41–80 km 80+ km	Distance	Increasing distances to hunting area lessens accessibility, limiting hunting opportunities and leading to smaller annual goose bags
Do you have a hunting dog?	Categorical No Yes	Dog	Ownership of one or more hunting dogs considered as substantial commitment (exercising dogs), increases number of days out hunting leading to greater success and higher annual goose bags
Which hunting equipment did you use most often when goose hunting in 2013–2014?	Categorical None Either Both	Equipment	Cost and skill investment in specialist goose hunting equipment (e.g. goose decoys and/or calls). Potentially increases success of each hunting day and hence larger annual goose bags
When did you mostly go goose hunting in 2013–2014?	Categorical It varies Weekdays Weekends/holidays Every opportunity	When	If restricted by when can go hunting (e.g. weekends) lessens hunting opportunities and hence smaller annual goose bags

^a^First level given for each categorical variable was set as the base comparative level

**Table 3:** Survey questions considered as potential motivational predictor variables, as well as additional attitude questions analysed

Survey question	Response type and modelled levels^a^	Predictor variable	Comments and hypothesized impact
How important are the following statements for you when you go on hunting?	Categorical		Selected series of attitude statements about hunter motivations for going goose hunting
For the sake of the challenge	Neutral (3) Not important (1, 2) Important (4, 5)	Challenge	Agreement suggestive of greater motivation to go goose hunting and potentially higher annual goose bags
To manage and control the number of geese	Neutral (3) Not important (1, 2) Important (4, 5)	Control	Agreement indicative of readiness to shoot more geese, achieving higher annual goose bags
For the meat	Neutral (3) Not important (1, 2) Important (4, 5)	Meat	Agreement suggestive of greater motivation for goose hunting to provide meat, leading to larger annual goose bags
To get peace and quiet from a stressful everyday life	Neutral (3) Not important (1, 2) Important (4, 5)	Peace	Agreement suggestive of enjoyment of experience rather than focusing on number of geese shot, potentially resulting in lower annual goose bags
How many geese would you be happy to shoot in a day’s goose hunt?	Categorical 0–2 3–5 6+	Goal	Preference for higher goose bags per hunting event, hence higher annual goose bags
How important do you think it is that hunting helps control the size of large goose populations in Denmark?	Categorical Neutral (3) Not important (1, 2) Important (4, 5)		Not used in models to predict goose bag sizes
If the hunting season was opened for more hunting of geese, how much more are you willing to shoot compared to your current yield?	Categorical 10% 25% 50% 75% 100% and more		Not used in models to predict goose bag sizes.
How much are you willing to reduce your current yield if the hunt should be limited to protect a falling goose population?	Categorical 10% 25% 50% 75% Stop completely		Not used in models to predict goose bag sizes

^a^First level given for each categorical variable was set as the base comparative level, and numbers in brackets indicate score on 5 point Likert scale used for these survey questions (1 = not at all important to 5 = extremely important)

#### Data exploration

Prior to fitting and model selection, we undertook exploratory data analyses to check for outliers and relationships between predictor variables. Cleveland dotplots and boxplots were used to visualize outliers. Variance inflation factor (VIF) values were used to assess collinearity (Zuur et al. 2009). This initial data exploration indicated that there were a number of outliers: 7 responses were considered as improbable and excluded from our analyses (e.g. 236 geese shot in 2 days). Initial analysis of the relationship between goose bag sizes and number of goose hunting days indicated overdispersion of the data. Consequently, we used negative binomial generalized linear models (GLM) with log link functions to determine associations between the response variable and selected predictor variables (Zuur et al. 2009).

#### Model selection and validation

We followed the information theoretic (I-T) approach (Burnham et al. 2011) to specify and test a series of candidate models. We combined predictor variables to create a series of potential models, reflecting the hypothesized hunter characteristics that might influence goose bag sizes. We started by formulating and testing two factor candidate models, where number of goose hunting days was combined with each of the categorical predictors, (e.g. days + equipment). We ran these models individually to assess their impact and to test the effects of different hunter behavioural characteristics. We then combined all behavioural covariates to create a full model, as well as adding interactions between ‘days’ and ‘distance’ and ‘days’ and ‘when’. Interactions were included as these two categorical predictors may potentially influence the number of hunting days, hence overall goose bag sizes. We then specified a series of behavioural candidate models with selected predictors removed to see if they improved or detracted from the full model. Data for all behavioural models were taken from 856 respondents who completed all selected behavioural questions. Model validation was applied to our optimal model following standard protocols, plotting Pearson residuals versus fitted values and for covariates to visualize and identify distinct patterns (Zuur et al. 2009). A second set of models was formulated to combine the best behavioural models with our selected motivational predictors. These models were formulated and selected following the same procedure as used for the behavioural models. These combined models used data from 756 respondents, as not all respondents completed the full set of selected motivational questions. This set of models included the two best behavioural models, without motivational predictors, for valid comparisons. We evaluated all models comparing Akaike’s information criterion (AIC) and ΔAIC values.

$$ \Delta_{i} = {\text{AIC}}_{i} {-}{\text{AIC}}_{\hbox{min} } ,\;{\text{for}}\;i = 1, \, 2, \ldots , \, R. $$

We did not use a correction for AIC values (AICc), as AIC figures are known to converge with large sample sizes (Burnham et al. 2011). All analyses were done using statistical procedures available in R 3.3.0 (https://cran.r-project.org/) and using the MASS and ggplot2 packages (Venables and Ripley 2002; Wickham 2009).

## Results

### Respondent characteristics

Virtually all respondents were male, with only four female respondents (*n* = 962). The average respondent age was 52 years (SD ± 14.55, median = 53, min–max = 18–86). For respondents indicating how long they had been goose hunting (*n* = 803), the average respondent was a long-time hunter having hunted geese for 16 years (SD ± 13.71, min–max = 1–65).

### Overall goose bag

**Fig. 1:**
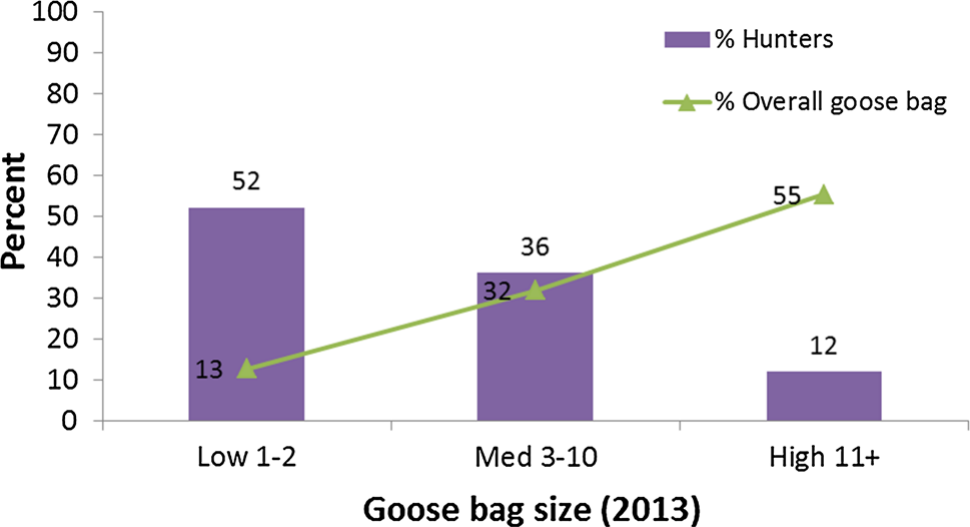
Proportion of hunters having shot geese by bag group and proportion of overall goose bag shot by each bag group during the 2013 hunting season (*n* = 9660)

For the 2013 hunting season, approximately 56 000 geese were shot by 9660 hunters who reported their hunting bags. The average hunter shot 6 geese in total (SD ± 14.11), but bag size distribution was heavily positively skewed (median = 2, min–max = 1–678). The majority (52%) of goose hunters only shot one or two geese during the course of the hunting season, accounting for 13% of the overall goose bag. Over half the annual reported goose bag was shot by a very small proportion of goose hunters (12%) (Fig. 1). Very similar patterns were seen for the 2014 and 2015 hunting seasons (Fig. S1, Appendix S2). The average hunter for this high bag group (11+ geese) shot 27 geese in total (SD ± 33.64, median = 18, min–max = 11–678). This analysis highlighted the impact of a small number of goose hunters on the overall annual goose bag.

#### Annual goose hunting activity

**Fig. 2:**
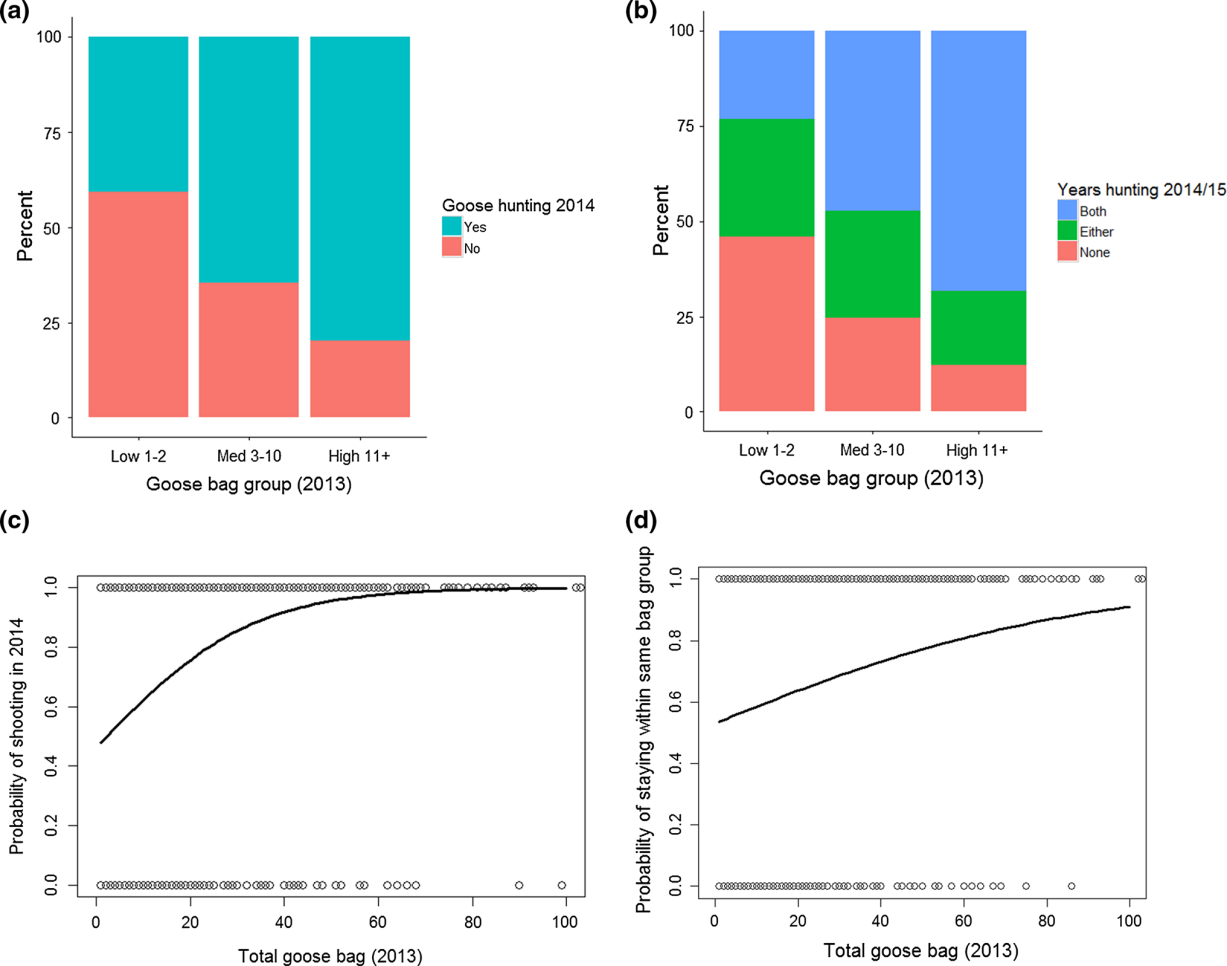
Goose hunting activity for 2014–2015 for all hunters who submitted goose bags in 2013 (*n* = 9660): **a** proportion of respondents who went on to shoot geese in 2014; **b** proportion of respondents who shot geese in consecutive years for the 2014 and 2015 hunting seasons; **c** predicted values (solid line) for binomial GLM representing probability of shooting in geese in subsequent 2014 hunting season. For each unit increase in bag size (1 goose), there would be an expected 7% increase in the odds of shooting geese in 2014 (*X*_1_^2^= 429.57, *p* < 0.001, odds ratio = 1.066*)*; **d** predicted values (solid line) for binomial GLM representing probability of hunters shooting geese staying in the same bag group in subsequent 2014 hunting season. For each unit increase in the bag size, there would be an expected 2% increase in the odds of staying in the same bag group (*X*_1_^2^= 67.94, *p* < 0.001, odds ratio = 1.022). Dots in **c** and **d** are observed values

Of hunters that submitted goose bag reports in 2013 (*n* = 9660), a large proportion (46%) did not go on to shoot geese in the following 2014 hunting season. However, there were considerable differences between goose bag groups. A very high portion of high bag hunters (11+ geese in 2013) went on to shoot geese in 2014 (80%), as well as shooting geese in consecutive years (2014 and 2015). The majority (59%) of low bag hunters (1–2 geese in 2013) did not shoot any geese in 2014, and 46% did not shoot geese in either 2014 or 2015 (Fig. 2a and b). A binomial GLM showed there was a positive relationship between total goose bag sizes in 2013 and the probability of shooting geese in 2014 (Fig. 2c). For those hunters who shot geese in 2013 and then went on to do so in 2014 (*n* = 5219), the majority (56%) remained in the same bag groups (1–2, 3–10, or 11+). The high bag group had the highest proportion not switching bag group (63%). As total bag size increased, the probability of staying in the same bag group increased, as indicated by a binomial GLM (Fig. 2d).

### Effect of goose hunting days

**Fig. 3:**
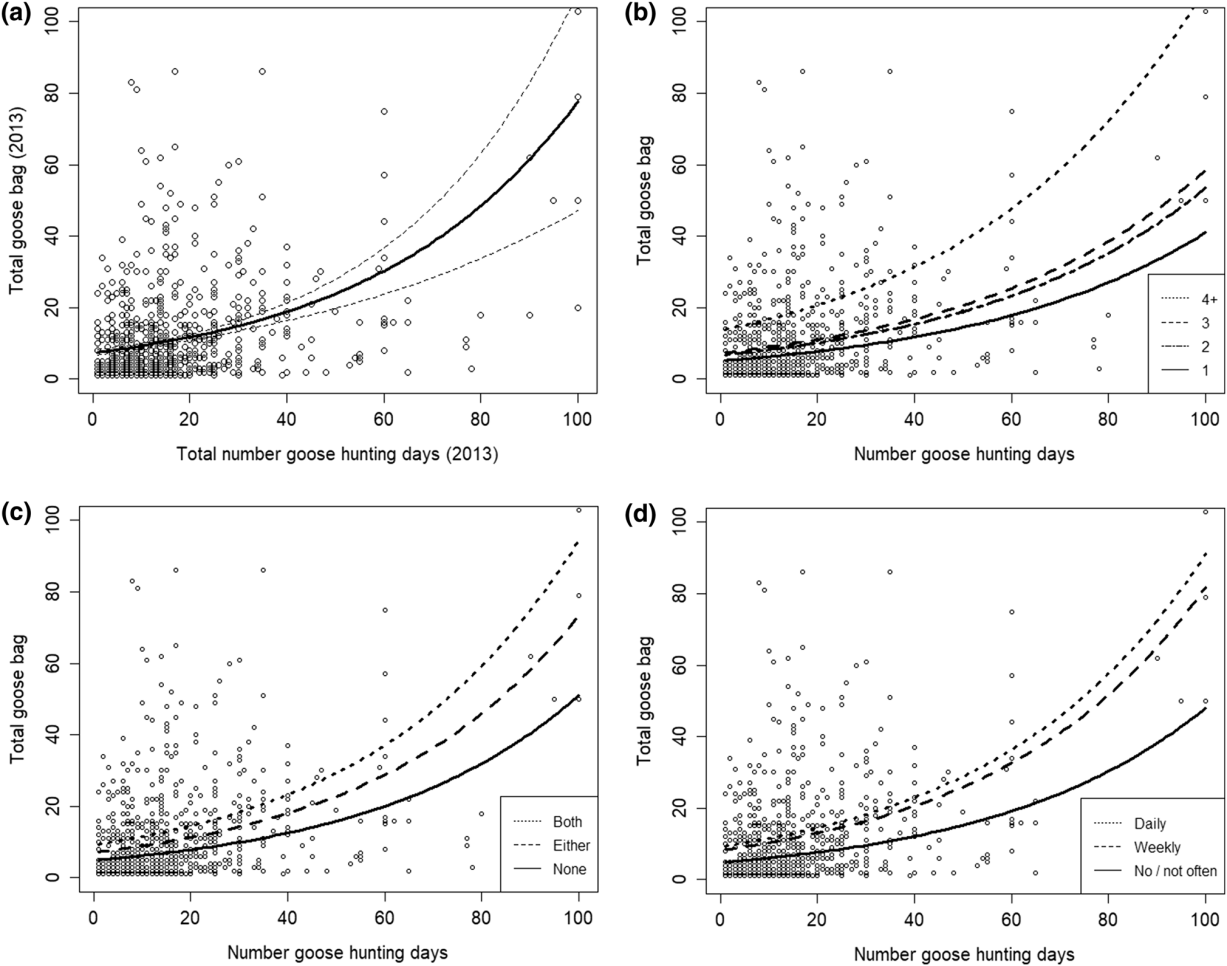
Observed respondent goose bags (dots) plotted against total number of goose hunting days. Plots show the combined effect of increasing goose hunting days, as well as the combined effect with each behavioural categorical predictor: **a** predicted values from the negative binomial model for the single variable ‘days’ have been added as a solid line, with dotted lines showing 95% confidence intervals. Predicted values for 3 best behavioural categorical predictors and levels are shown with lines as indicated: **b** days + areas; **c** days + equipment; **d** days + check

Obtained from survey data, the median total number of goose hunting days for survey respondents was 12 (*n* = 905), with the distribution positively skewed (min–max = 1–110, excluding outliers). A negative binomial GLM showed there was evidence for a positive relationship between the number of goose hunting days (as the only predictor) and total goose bags (*b* = 0.02, *z* = 10.08, *p* < 0.001) (Fig. 3a). However, this single predictor variable only explained 10.5% of goose bag variance, suggesting that other factors may contribute to and predict goose bag sizes. Some respondents achieved high goose bags over several days (e.g. one respondent shot 27 geese in 4 days), whilst other respondents indicated many goose hunting days but achieved relatively low goose bags. These were all considered feasible outcomes but raised questions about (1) why some respondents appeared to be more successful; (2) whether some respondents were actually goose hunting each time?

### Effects of hunter characteristics

#### Behavioural predictors

The optimal behavioural model was found to be one which included all selected behavioural predictors, as well as specified interactions. This model (AIC = 5638.5; Table 4), explaining 30.7% of goose bag variance (parameter estimates in Table S1, Appendix S2). Other candidate models with single and combinations of behavioural predictors did not improve model fits. ΔAIC values were considerably larger than those needed to support them as plausible competing models, ΔAIC < 14 (Burnham et al. 2011). Comparing AIC values for these candidate models (Table 4) indicated that the three most influential behavioural predictors when individually combined with ‘days’ were (1) having access to multiple hunting ‘areas’ (AIC = 5727.7); (2) use of ‘specialist equipment’ (AIC = 5777.6) and (3) ‘checking’ for geese in hunting areas (AIC = 5781.3). These three predictors all had positive effects on bag sizes. We visualized predicted values for these categorical levels in models including number of goose hunting days (Fig. 3b–d). Full set of plotted predicted values for all behavioural variables are provided in Fig. S2, Appendix S2.

**Table 4:** AIC values for all formulated behavioural models (*n* = 856)

Models	AIC	ΔAIC	ED^a^ (%)
Days + area + check + distance + dog + equipment + when + days × distance + days × when	5638.5	0	30.7
Days + area + check + distance + dog + equipment + when	5657.9	19.4	28.2
Days + area + check + distance + equipment + when (minus dog)	5658.5	25	28.0
Days + area + check + dog + equipment + when (minus distance)	5664.7	26.2	27.1
Days + area + check + distance + dog + equipment (minus when)	5672.4	33.9	26.5
Days + area + check + equipment (minus distance, dog and when)	5685.5	52	24.7
Days + areas + distance + when (minus check, dog and equipment)	5697.6	64.1	24.1
Days + areas	5727.7	94.2	20.4
Days + check + dog + equipment	5740.1	101.6	19.6
Days + equipment	5777.6	144.1	15.7
Days + check	5781.3	147.8	15.3
Days + when	5796.6	163.1	14.1
Days + dog	5813.2	174.7	12.1
Days + distance	5816	182.5	12.2
Areas	5816.7	183.2	12.0
Days	5827.5	194	10.5
Equipment	5886.2	252.7	4.9
Check	5887.5	254	4.7
When	5891.7	258.2	4.5
Distance	5919.6	281.1	1.6
Dog	5920.3	281.8	1.1

^a^Explained deviance

#### Motivational predictors

Adding all motivational predictors to the full behavioural model without interactions (AIC = 5054.4) improved this model (AIC = 4948.2), based on comparative AIC values (Table S2, Appendix S2). Further model tests showed that the motivational predictors ‘meat’ and ‘peace’ were not statistically significant, whilst the predictor ‘challenge’ was only marginally significant (*p* < 0.1). Our analyses indicated that the two most influential motivational predictors were (1) whether respondents considered their hunting activity important for managing and controlling goose populations, or (2) whether they were motivated to shoot a high number of geese each hunting event (Fig. S3, Appendix S2). Adding these two predictors to the optimal behavioural model, including interactions, gave the lowest AIC value (AIC = 4935.5), explaining 38.9% of goose bag variance. Although motivational characteristics are indicative of higher goose bags, most respondents (71%) were satisfied with only shooting 0–2 geese each time; a much smaller proportion (6%) desired 6+. Furthermore, most respondents (59%) also indicated managing geese was not an important motivational factor to go hunting, with only 10% indicating it was important.

### Other hunting activity

**Fig. 4:**
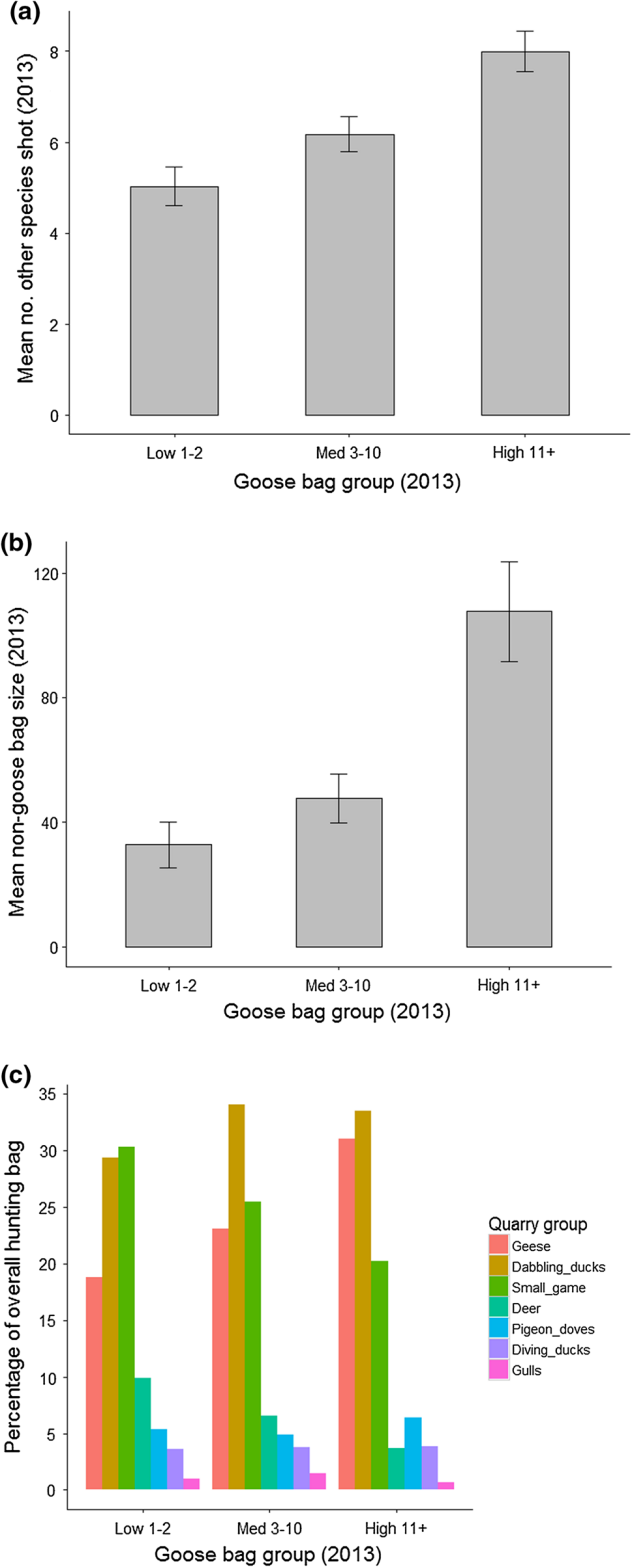
Other huntable species shot by survey respondents (*n* = 905) in 2013: **a** mean number of non-goose species shot by respondent groups, **b** mean number of non-goose individuals shot by respondent groups, **c** proportion of overall hunting bag for individuals shot within species groupings, by respondent group

Analysis of survey respondents’ 2013 hunting bag records showed that the average respondent shot several huntable species other than geese (mean = 7, SD ± 3.94, median = 6, min–max = 1–22), but there were differences between goose bag groups (Fig. 4a). The average respondent’s overall non-goose bag for 2013 was 65 individuals (SD ± 107.35), but the non-goose bag distribution was positively skewed (median = 30, min–max = 0–1181). Comparing respondent groups showed that those with a higher goose bags shot a greater variety of species and shot many more individuals (Fig. 4a and b). Further analysis of the number of individuals shot within species groupings showed that there were slight differences between respondent groups in the composition of their hunting bags (Fig. 4c). It is evident that most respondents did not just shoot geese and had much broader hunting interests.

### Managing geese and willingness to alter hunting activity

**Fig. 5:**
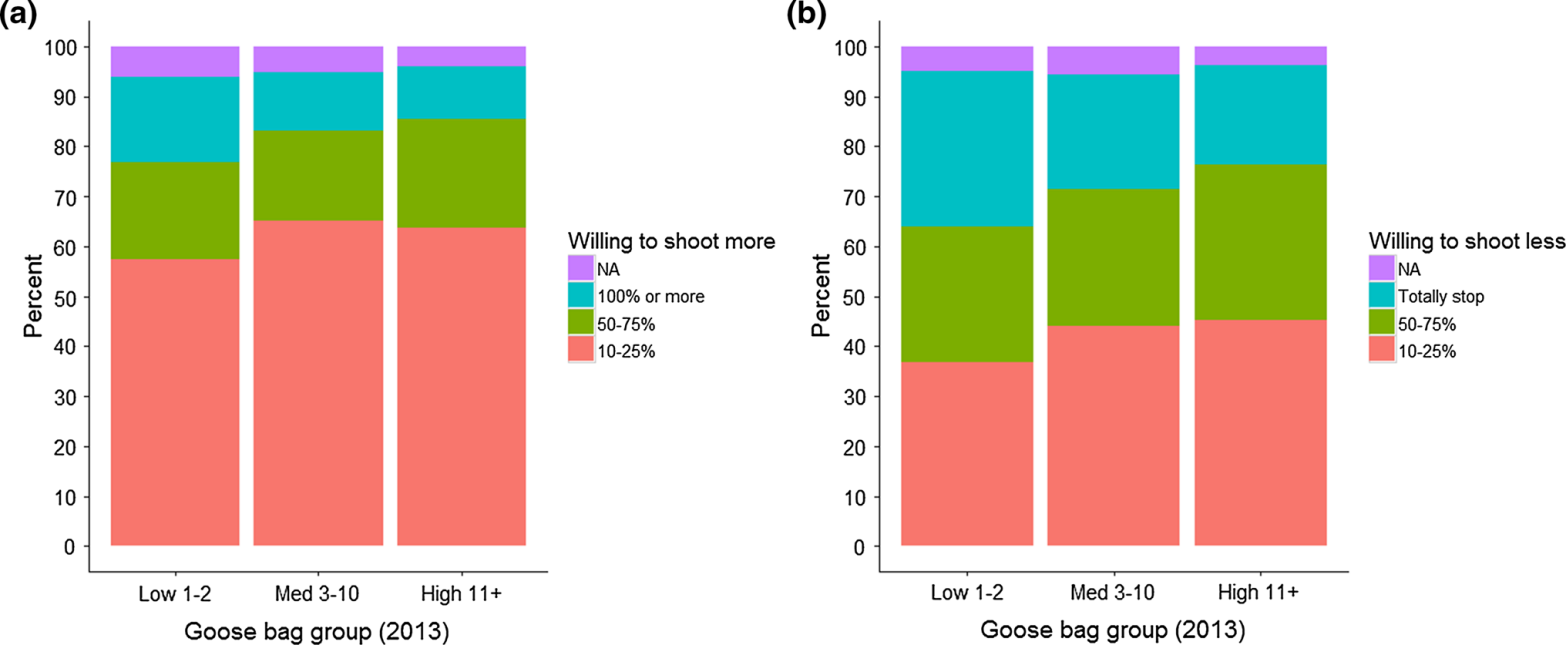
Proportion of respondents willing to alter how many geese they shoot from current goose bag sizes (*n* = 905): **a** willingness to increase goose bag if hunting of geese was opened and freely permitted; **b** willingness to reduce goose bag if hunting of geese was restricted to protect a falling goose population

The majority (62%) of respondents indicated that they considered hunting an important tool to control the size of large goose populations. A higher proportion of respondents (73%) in the high bag group considered hunting an important tool in comparison to the other bag groups. Using Pearson’s Chi-square test showed this was a significant association, but Cramér’s *V* test indicated it was weak (*X*^2^ = 26.02, *p* > 0.05, d.f. = 4, *ϕc *= 0.1). 62% of respondents indicated that they would only be willing to increase the number of geese they shot by 10 or 25%, in comparison to their current goose bag. Using Pearson’s Chi-square test showed there was no significant association between respondent bag groups and their willingness to shoot more geese (*X*^2^ = 7.99, *p* > 0.05, d.f. = 4) (Fig. 5a). Using the 2013 bag size for each respondent and the percentage increase they indicated, we calculated an overall 35% uplift in the annual goose bag, if respondents were requested to shoot more. In order to protect a declining goose population, 25% of all respondents would be willing to totally stop, whilst a further 28% would be willing to reduce their goose bag by at least 75%. A Pearson’s Chi-square test showed there was a significant association between respondent bag groups and their willingness to shoot less geese (*X*^2^ = 11.31, *p* < 0.05, d.f. = 4); however, Cramér’s *V* indicated this association was weak (*ϕc* = 0.1) (Fig. 5b). Based on each respondent’s 2013 bag size and the percentage decrease they indicated, we calculated an overall 43% reduction in the annual goose bag, if respondents were requested to shoot less.

## Discussion

Our study has shown that the majority of Danish hunters that shot geese did so only on a casual basis. Most hunters tended to shoot just one or two geese during the course of a hunting season and were less likely to shoot geese every year. These low bag hunters only had a small impact on the number of geese shot, whilst the majority of geese were shot by a comparatively small proportion of hunters. The focus of the ISMP for the pink-footed goose was initially on increasing harvests, as the size of the population continued to grow. This guided our interest in determining what characteristics enabled some hunters to achieve higher goose bags. We have shown there is a positive relationship between the number of hunting days and bag size. This echoes findings of a study amongst duck hunters in the US (Haugen et al. 2015), but our data also showed considerable variation. Some respondents achieved high goose bags whilst indicating these were shot over just a few hunting days. Unlike the US, Denmark has no daily bag limits. Our analyses did indicate that 3 behavioural characteristics may enhance the success of hunting when a hunter does go out hunting, leading to higher bag sizes: access to multiple hunting areas, using specialist equipment (e.g. decoys and/or goose calls) and actively checking for geese prior to hunting. These characteristics indicate that investment and commitment is needed to be an effective goose hunter. Waterbird hunting is known for its high degree of specialization, in comparison to other hunting types (Miller and Graefe 2000). For wildlife managers endeavouring to manage rapidly growing goose populations, specialization and use of hunting as a management tool has many implications. Our study has provided useful insights regarding three broad aspects that are particularly relevant where recreational hunting is to be employed and increased harvest rates required (1) the ability and effectiveness of recreational hunters to attain required harvest bags, (2) their willingness to alter their hunting activity and (3) act as responsible stewards, i.e. undertaking actions specifically to care for a valued resource (Bruskotter and Fulton 2012).

Certainly higher harvests can be attained using regulatory mechanisms such as extending hunting seasons. Where this has been shown to be the case (Madsen et al. 2016), it was not known whether any particular group of hunters contributed more than others to the increased harvest (e.g. was it new entrants, a general increase in individual bag sizes or a small group of hunters shooting many more)? As the majority of hunters, in our study, were casually involved in goose hunting, would they have sufficient access, knowledge and skills to hunt geese effectively and increase overall goose harvests? We have shown that attaining higher goose bags requires a degree of specialization. Specialization is viewed as progressive development over time from novice to expert (Scott and Shafer 2001; Kuentzel and Heberlein 2006). It has also been shown that participation in hunting can decrease if there are constraints (e.g. limited access to hunting areas or needing specialist equipment) (Barro and Manfredo 1996). Devoting the time, gaining access (hunting rights) and the purchase of specialist equipment may pose considerable barriers for casual hunters or new entrants. These factors may impede attaining the necessary proficiency in goose hunting and the ability of these hunters to increase their harvests without detrimental consequences (e.g. wounding). This latter point has significance for societal acceptance if recreational hunting is to be used to manage wildlife populations, such as geese.

The majority of respondents in our study agreed that hunting has an important role in managing goose populations. Furthermore, our study has shown that most hunters would be willing to increase, as well as decrease their hunting effort, potentially leading to large changes in overall annual goose harvests. However, our results, somewhat surprisingly, indicated that motivational factors did not have much influence in determining goose bag sizes. This seems to reflect that motivations to go hunting span a variety of experience preferences (Schroeder et al. 2006; Harper et al. 2012), rather than purely consumptive motives that have often been associated with hunters in the past (Wam et al. 2013). We have shown that most respondents were less focused on achieving high daily goose bags and likely to be satisfied by shooting, at most, a couple of geese each time they hunted. For the majority of respondents, managing geese was not an important motivational factor to go goose hunting. Furthermore, respondents in our study had broad hunting interests and were not solely focused on shooting geese but participated in several forms of hunting (e.g. duck, small game and deer). These findings suggest that, although respondents indicated their willingness to alter their goose hunting effort, personal circumstances, hunting preferences as well as other external factors may mitigate these intentions. It has been shown that social pressures and the perceived ease or difficulty of performing a behaviour can have a significate effect on intentions in specific hunting contexts (hunting species limited in time and place) (Hrubes et al. 2001; Shrestha et al. 2012). Hence, constraints perceived by hunters (e.g. access to quality hunting areas or lacking specialized hunting skills/equipment) may hinder future participation.

There are potential risks and unintended consequences of actively promoting and pursuing higher harvests, such as the wounding of geese. This may be related to increased exposure to less experienced or new goose hunters. This has implications for wildlife managers and hunting organizations, if recreational hunting is to be employed as a management tool. Increasingly, as society changes (e.g. urbanization) and values shift (e.g. towards a mutualist orientation), hunting comes under greater scrutiny from animal ethics points of view and sections of society hold more negative attitudes towards hunting (Gamborg and Jensen 2017). This was a noted ethical concern for the ISMP of the pink-footed goose (Madsen et al. 2017). Mitigating measures can be successfully employed to minimize wounding, for example hunter education, skill development and promoting beneficial hunting practices (e.g. use of decoys and goose calls) (Noer et al. 2007). There is evidence of an increase in the efficiency amongst Danish goose hunters, as wounding has declined despite increasing harvests (Clausen et al. 2017). Our study has indicated that specialization is associated with more effective goose hunting practices and this may have been a major contributor to the decline. Thus, it would seem if hunting is to be successfully employed as a management tool, wildlife managers and hunting organizations need to consider facilitating the development of skills and beneficial practices amongst targeted groups of recreational hunters. Hunters are themselves increasingly recognizing the multi-functionality of their recreational pastime and hunting ethics continue to evolve with hunters self-imposing socially, aesthetically and morally defensible ways of hunting (Kaltenborn et al. 2013; Gamborg et al. 2017; von Essen 2018). Consideration should be given to how best to promote and instil suitable hunting practices as higher social standards dictate why, how and in what circumstances particular hunting practices are acceptable to manage wildlife populations.

## Conclusions

The use of recreational hunters to manage wildlife populations raises many practical challenges and ethical issues for wildlife managers. There is growing recognition that human components and societal values need to be explicitly accounted for in adaptive management (Enck et al. 2006; Johnson et al. 2015; Schroeder et al. 2017). Our study was focused on the management of a rapidly increasing goose population and the potential of recreational hunters to achieve management goals that are both ecologically and socially desirable. We have identified several behavioural characteristics that typify effective goose hunting practices, suggesting a degree of specialization is necessary to increase goose harvests, as well as mitigating animal welfare issues (e.g. wounding). Our study also suggests that most goose hunters, although recreational, do consider they have stewardship role and would be willing to adjust their hunting effort. However, in our study most hunters only shot one or two geese, and not every year, and had a limited impact on overall goose harvests. The challenge for wildlife managers is to engage and influence such a relatively large section of the hunting community as part of an adaptive harvest management regime. This is important, if hunters interest in one form of hunting (e.g. goose hunting) is casual, and their hunting preferences, circumstances and perceived constraints hinder or conflict with the achievement with management objectives. If recreational hunters are to be used as a management tool, wildlife managers and hunting organizations will need to consider how best to facilitate skill development, hunting practices and socially legitimate hunting ethics to foster the stewardship role of hunting. We conclude that it is incumbent on wildlife managers to recognize and deal with the internal factors (e.g. skill development and hunter satisfaction) and external influences (e.g. animal welfare concerns) to alleviate potential tensions in the multi-functionality of hunting as a legitimate and accepted recreational past-time and management tool.

## Electronic supplementary material

Below is the link to the electronic supplementary material.

Supplementary material 1 (PDF 757 kb)
