# Supplementary material for: Managing geese with recreational hunters?
Source: Ambio. 2018 Jul 3;48(3):217–29. doi: 10.1007/s13280-018-1070-7 (PMC6374222; doi:10.1007/s13280-018-1070-7)
Supplement: Supplementary file 1 — Supplementary material 1 (PDF 757 kb) [file 13280_2018_1070_MOESM1_ESM.pdf]

Ambio

Electronic Supplementary Material
This supplementary material has not been peer reviewed

Title: **Managing geese with recreational hunters?**

Appendix S1: Methods

Table S1. Questions used from Danish goose hunters' survey conducted in September 2014.

No.	Question	Response
9	Do you have a hunting dog?<br>(Tick only one box!)	Multiple choice question:<br>I. No<br>II. Yes, one<br>III. Yes, more than one
12	Which of the following answers best describes when you mostly went waterbird hunting in 2013-14?<br>(Tick only one box!)	Multiple choice question (random rotation):<br>I. On weekdays<br>II. Weekends and / or holidays<br>III. On vacation<br>IV. It varies<br>V. Every opportunity
13	Approximately how many days were you out hunting and you shot geese during 2013-14 hunting season?<br>Enter the number of days:	Open question: number of days
14	How often were you out goose hunting WITHOUT shooting geese in 2013-14?<br>Enter the number of days:	Open question: number of days
18	What was the distance from your home to your most often used goose hunting area in 2013-14?<br>(Tick only one box!)	Multiple choice question:<br>I. 0 km<br>II. 1-10 km<br>III. 11-20 km<br>IV. 21-40 km<br>V. 41 - 60 km<br>VI. 61 – 80 km<br>VII. 81 – 100 km<br>VIII. 101 – 150 km<br>IX. 151 – 200 km<br>X. Over 200 km
20	How many goose hunting areas do you have access to, including your most often used area?<br>(Tick only one box!)	Multiple choice question:<br>I. 1<br>II. 2<br>III. 3<br>IV. 4<br>V. 5 or more
27	Which hunting equipment did you use most often when goose hunting in 2013-14?	Multiple choice question (random rotation):<br>I. Decoys<br>II. Hide / camouflage<br>III. Goose call<br>IV. Boat or barge<br>V. 'Lay out blinds'<br>VI. None of the others<br>VII. Other (specify)
30	About how many years have you been going goose hunt?<br>Enter the number of years.	Open question: number of years

37	How often do you or your hunting partners check if geese are in your hunting area?<br>(Tick only one box!)	Multiple choice question:<br>I. Every day<br>II. A few times a week<br>III. Every week<br>IV. Not so often<br>V. Don’t check
43	How important are the following statements for you when you go on hunting? Please indicate on a scale between 1 and 5.<br>1 = Not at all important<br>5 = Extremely important	Matrix question: 5 point Likert scale<br>I. It is exciting to shoot geese<br>II. For the sake of the challenge<br>III. To get peace and quiet from a stressful everyday life<br>IV. For the meat<br>V. For the nature experience<br>VI. To work with hunting dogs<br>VII. To manage and control the number of geese<br>VIII. It is cheaper than other types of hunting<br>IX. It is more accessible / practical than other types of hunting
44	How many geese would you be happy to shoot in a day's goose hunt?<br>(Tick only one box!)	Multiple choice question:<br>I. 0 geese<br>II. 1-2 geese<br>III. 3-5 geese<br>IV. 6-10 geese<br>V. 11-20 geese<br>VI. 21 - 30 geese<br>VII. 31 - 40 geese<br>VIII. Over 40 geese
53	How important is it that hunting is used as a tool for controlling the size of large goose stocks in Denmark? Please indicate on a scale between 1 and 5.	Multiple choice question: 5 point Likert scale<br>I. 1 = Not at all important<br>II. 5 = Extremely important 1-2 geese
54	If hunting was opened to allow for more geese to be shot, how much more would you like to shoot compared to your current yield?<br>(Tick only one box!)	Multiple choice question:<br>I. 10%<br>II. 25%<br>III. 50%<br>IV. 75%<br>V. 100% or more
55	How much are you willing to reduce your current yield if the hunt is to be limited to ensure a falling goose stock?<br>(Tick only one box!)	Multiple choice question:<br>I. 10%<br>II. 25%<br>III. 50%<br>IV. 75%<br>V. Totally stop

Appendix S2: Results

Fig. S1. Proportion of hunters having shot geese by bag group and proportion of overall goose bag shot by each bag group during the: a) 2014 hunting season (n = 13 200), b) 2015 hunting season (n = 13 400)

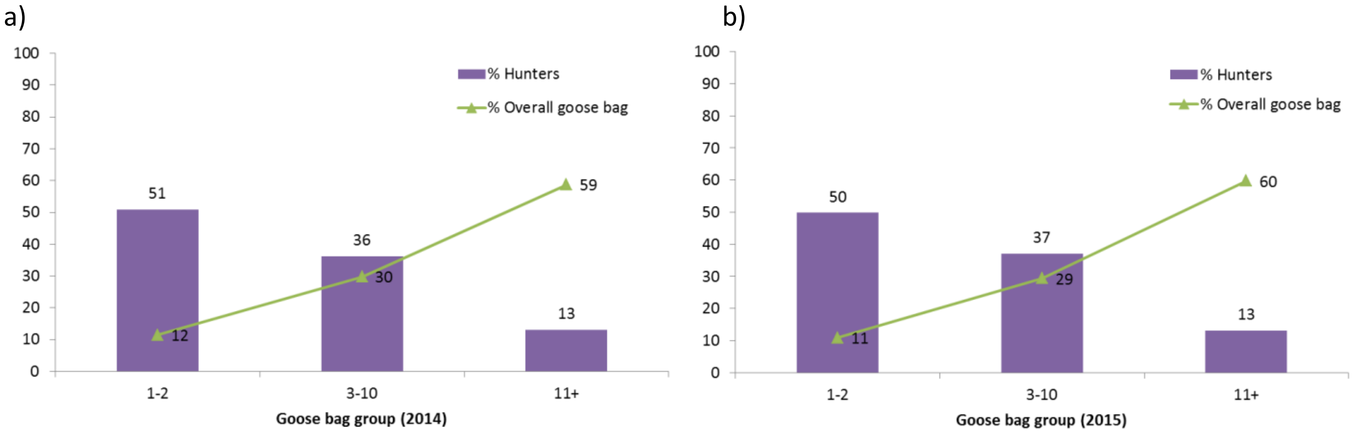

Table S1: Effects for predictors from log link function negative binomial generalized linear model for optimal behavioural model: Days + area + check + distance + dog + equipment + when + days x distance + days x when (AIC = 5638.5)

Effects	Level	B	SE	z-value	P
Intercept		0.989	0.143	6.931	<0.001
Days	Continuous	0.033	0.005	6.627	<0.001
Dog	No	0			
	Yes	0.0887	0.080	1.104	
Area	1	0			
	2	0.162	0.084	1.916	<0.1
	3	0.225	0.094	2.381	<0.05
	4+	0.732	0.099	7.419	<0.001
Equipment	None				
	Either	0.227	0.088	2.580	<0.01
	Both	0.406	0.080	5.065	<0.001
Checking	No / not often	0			
	Weekly	0.305	0.081	3.789	<0.001
	Daily	0.458	0.105	4.350	<0.001
When	It varies				
	Weekdays	-0.027	0.155	-0.175	
	Weekends/holidays	-0.312	0.132	-2.366	<0.05
	Every opportunity	0.439	0.124	3.540	<0.001
Distance	0-10km	0			
	11-40km	-0.048	0.117	-0.409	
	41-80km	0.723	0.175	4.124	<0.001
	80+km	0.005	0.153	0.031	
Days x when	Days*it varies	0			
	Days*weekdays	-0.006	0.007	-0.849	
	Days*weekends/holidays	0.007	0.007	0.956	
	Days*every opportunity	-0.018	0.006	-3.089	<0.001
Days x distance	Days*0-10km	0			
	Days*11-40km	-0.006	0.005	-1.277	
	Days*41-80km	-0.043	0.010	-4.019	<0.001
	Days*80+km	0.004	0.008	0.565	

Fig. S2. Observed respondent goose bags (dots) plotted against total number of days goose hunting. Plots show the combined effect of increasing goose hunting days with each behavioural categorical predictor. Predicted level values are shown with lines as indicated: a) days + areas; b) days + equipment; c) days + check; d) days + dog; e) days + when; f) days + distance.

a)

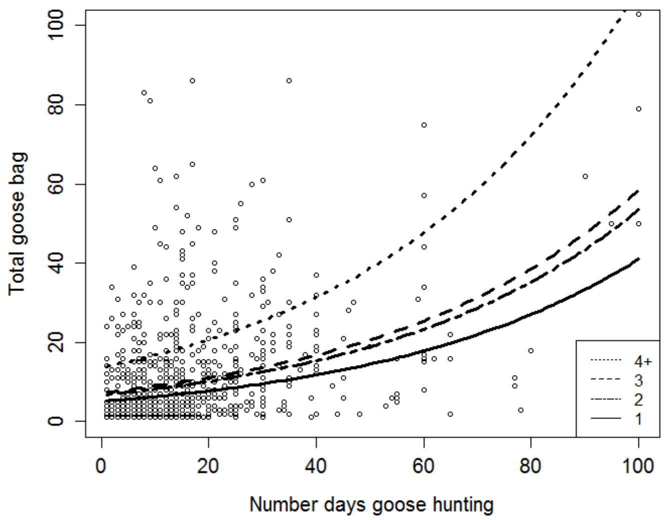

b)

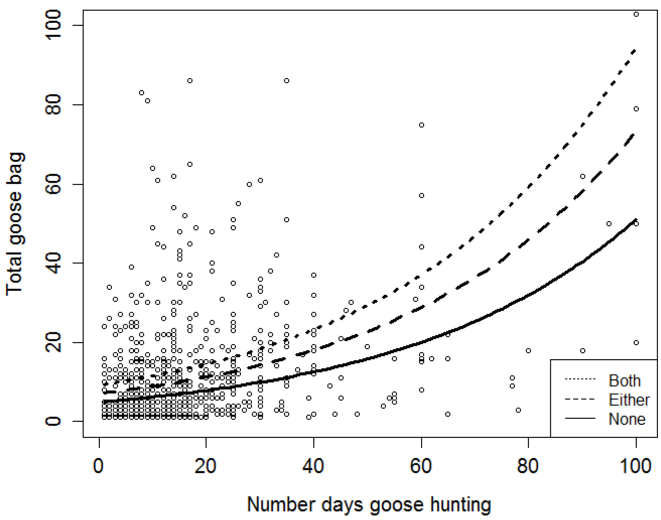

c)

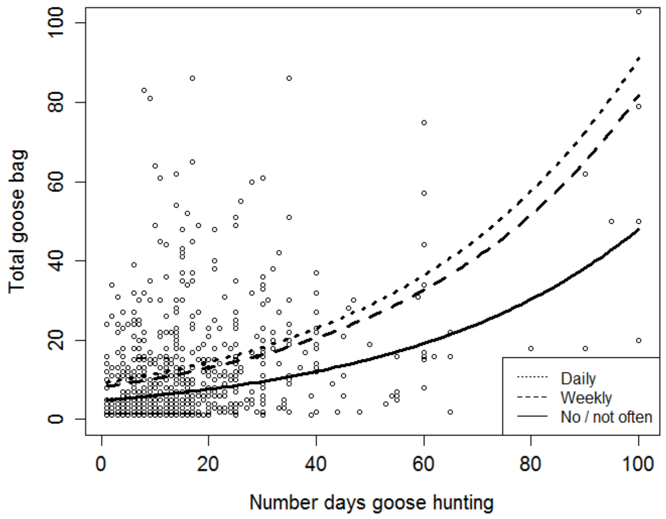

d)

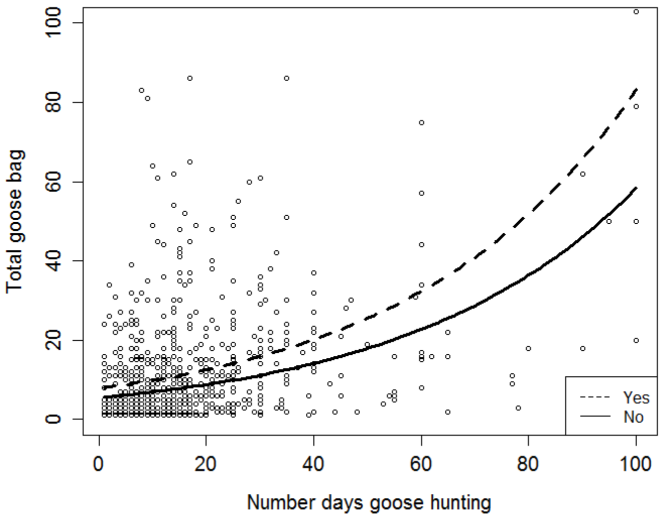

e)

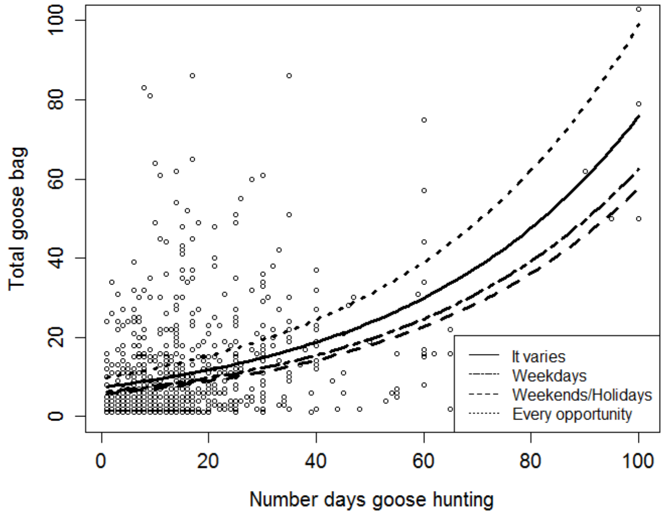

f)

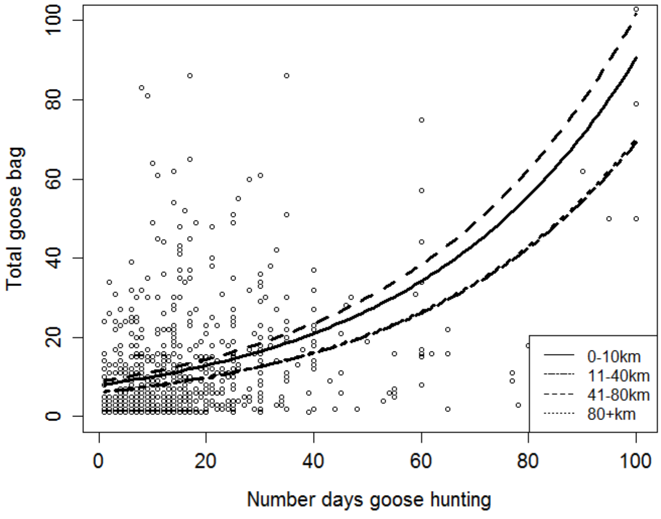

Table S2. AIC values for all formulated combined motivational and behavioural models (n = 756).

Models	AIC	ΔAIC	ED^{+} (%)
Days + areas + check + distance + dog + equipment + when + days x when + days x distance + goal + control	4935.5	0	38.9
Days + areas + check + distance + dog + equipment + when + challenge + control + goal (minus meat & peace)	4944.6	9.1	37.5
Days + areas + check + distance + dog + equipment + when + control + goal (minus challenge¸ meat & peace)	4946.8	11.3	37.0
Days + areas + check + distance + dog + equipment + when + challenge + control + goal + meat + peace	4948.2	12.7	37.8
Days + areas + check + distance + dog + equipment + when + goal (minus challenge + control + meat + peace)	4970.5	35	34.6
Days + equip + check + dog + area + days x when + days x distance	5030.5	95	30.1
Days + equip + check + dog + area + days + when	5054.4	118.9	26.2

$^{+}$ = Explained deviance

Fig. S3. Observed respondent goose bags (dots) plotted against total goose hunting days. Plots show the combined effect of increasing goose hunt days with the two most influential motivational predictors. Predicted level values are shown with lines as indicated: a) days + control; b) days + goal.

a) b)

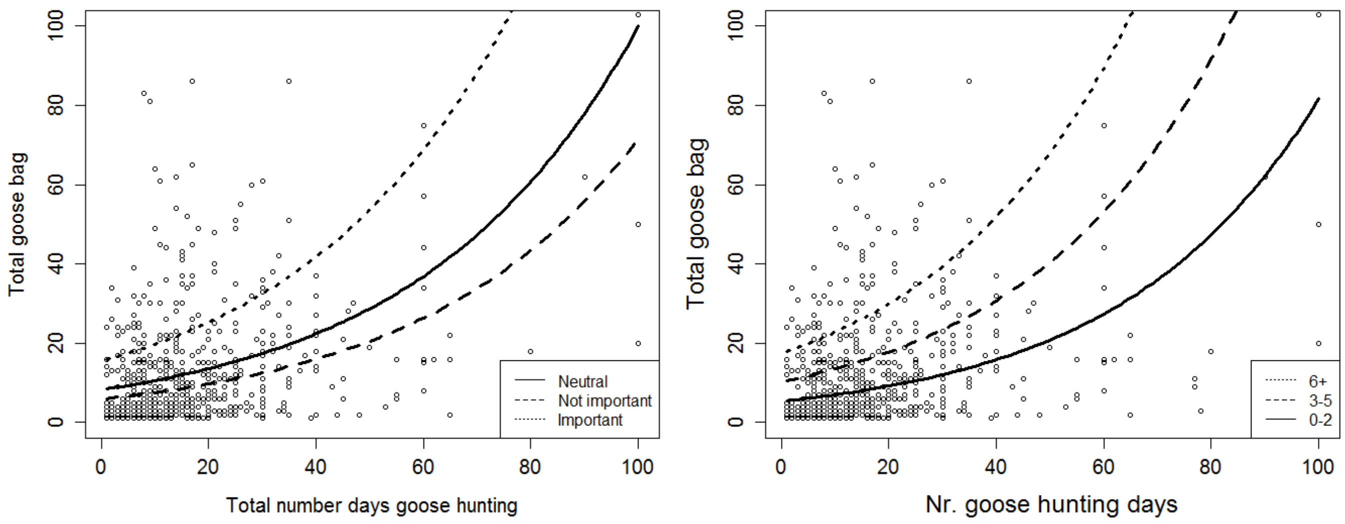

Table S3: Effects for predictors from log link function negative binomial generalized linear model for optimal behavioural and motivational model: Days + areas + check + distance + dog + equipment + when + days x when + days x distance + goal + control (AIC = 4935.5)

Effects	Level	B	SE	z-value	P
Intercept		0.928	0.166	5.584	<0.001
Days		0.038	0.005	7.640	<0.001
Dog	No	0			
	Yes	0.072	0.083	0.872	
Area	1	0			
	2	0.146	0.087	1.677	<0.1
	3	0.225	0.096	2.330	<0.05
	4+	0.624	0.101	6.137	<0.001
Equipment	None	0			
	Either	0.165	0.092	1.801	<0.1
	Both	0.319	0.083	3.840	<0.001
Checking	No / not often	0			
	Weekly	0.203	0.083	2.445	<0.05
	Daily	0.248	0.110	2.255	<0.05
When	It varies	0			
	Weekdays	-0.029	0.176	-0.164	
	Weekends/holidays	-0.238	0.132	-1.799	<0.1
	Every opportunity	0.371	0.124	2.976	<0.05
Distance	0-10km	0			
	11-40km	0.097	0.123	0.079	
	41-80km	0.718	0.175	3.919	<0.001
	b80+km	0.115	0.154	0.745	
Control	Neutral	0			
	Not important	-0.133	0.082	-1.614	
	Important	0.358	0.117	3.050	<0.05
Goal	0-2	0			
	3-5	0.498	0.080	6.203	<0.001
	6+	0.825	0.132	6.233	<0.001
Days x when	Days*it varies	0			
	Days*weekdays	-0.008	0.008	-0.937	
	Days*weekends/holidays	-0.001	0.007	-0.090	
	Days*every opportunity	-0.018	0.006	-3.145	<0.001
Days x distance	Days*0-10km	0			
	Days*11-40km	-0.009	0.005	-1.687	<0.1
	Days*41-80km	-0.039	0.011	-3.549	<0.001
	Days*80+km	0.004	0.008	0.516	
